# Body Mass Index in Multiple Sclerosis: Associations with CSF Neurotransmitter Metabolite Levels

**DOI:** 10.1155/2013/981070

**Published:** 2013-09-24

**Authors:** Manolis Markianos, Maria-Eleftheria Evangelopoulos, Georgios Koutsis, Panagiota Davaki, Constantinos Sfagos

**Affiliations:** Department of Neurology, Eginition Hospital, Athens University Medical School, Vassilissis Sophias 74, 11528 Athens, Greece

## Abstract

Body weight and height of patients with relapsing-remitting multiple sclerosis (RRMS) or clinically isolated syndrome suggesting MS (CIS) in the age range 18 to 60 years (154 males and 315 females) were compared with those of subjects (146 males and 212 females) free of any major neurological disease. In drug-free patients, CSF levels of the metabolites of noradrenaline (MHPG), serotonin (5-HIAA), and dopamine (HVA), neurotransmitters involved in eating behavior, were estimated in searching for associations with body mass index (BMI). Statistical evaluations were done separately for males and females. Lower BMI was found in female MS patients compared to female controls, more pronounced in RRMS. BMI was not associated with duration of illness, smoking, present or previous drug treatment, or disability score. Body height showed a shift towards greater values in MS patients compared to controls. Patients in the lower BMI quartile (limits defined from control subjects) had lower 5-HIAA and HVA compared to patients in the upper quartile. The results provide evidence for weight reduction during disease process in MS, possibly related to deficits in serotoninergic and dopaminergic activities that develop during disease course, resulting in impairments in food reward capacity and in motivation to eat.

## 1. Introduction

Multiple sclerosis (MS) is an autoimmune demyelinating disease of the central nervous system. Although viral and genetic factors have been postulated to trigger the autoimmune process, the pathogenetic mechanisms regulating the disease course remain unknown. Increasing amount of evidence suggests that physical comorbidities as well as adverse health factors might affect the disease course [1].

The impact of nutrition and diet in the aetiology and management of MS has been investigated in a large number of studies [2–6], but with only a limited number reported on body mass index (BMI) in MS. Ghadirian et al. [3] found an inverse association between high BMI and the risk of MS. The difference in BMI was significant for the whole sample and for female MS patients compared to controls, while it did not reach significance for males. Formica et al. [7] reported a lower BMI in women with MS compared to female controls, and Nortvedt et al. [8] reported significantly lower BMI of MS patients (75% females) compared to the general population. Similarly, Alschuler et al. [9] reported lower BMI in a large number (*n* = 597) of patients with MS in relation to general population. On the other hand, Khurana et al. [10] reported an increased prevalence of overweight and obesity in a large sample of older veterans with MS (mean age 59.6 ± 11.9), predominantly males (86.7%). In their study of mobility problems in MS patients from five European countries, Pike et al. [11] reported a mean BMI of 23.9 for a sample of 3572 patients (36% males) over 18 years of age, 4.6 mean number of years since diagnosis. Munger et al. [12] found increased risk for MS in females who have been obese at adolescence but no association of disease risk with adult BMI. Similarly, Hedstrom et al. [13] reported no differences in BMI between MS patients and controls, but they found that subjects who reported body weight at age 20 that gave a BMI over 25 had higher risk for developing MS. Overweight in adolescence was considered as a predisposing factor in developing MS, although at disease onset, patients seem to have normal weight or even to be underweighted, especially female patients. This may indicate that not being overweight at adolescence but losing weight after adolescence may be the predisposing factor. At any case, it is an open question if BMI is normal at disease onset and reductions develop during disease course.

Regulation of food intake is a highly complex process involving many hormonal and neuropeptide pathways, including monoamine neurotransmitters [14]. Increases in dopamine (DA) signaling promote feeding, with leptin and insulin inhibiting and ghrelin activating dopamine neurons, while too much dopamine signaling seems to inhibit feeding [15]. Experimental models with dopamine deficient mice have demonstrated that these animals are aphagic and do not survive unless DA synthesis is restored [16, 17]. Dopamine seems to energize feeding and to reinforce food-seeking behavior, while food reward elevates dopamine levels [18].

In a recent study [19], we reported that subjects in the upper BMI quartile show higher levels of the serotonin and dopamine metabolites 5-hydroxyindoleacetic acid (5-HIAA) and homovanillic acid (HVA) than subjects in the lower and middle quartiles, indicating an association of higher serotonin and dopamine turnover with overweight. No differences were found in the levels of the noradrenaline metabolite methoxyhydroxyphenylglycol (MHPG). We had previously reported changes in CSF MHPG, 5-HIAA, and HVA levels in MS patients during disease course, possibly relevant regarding BMI [20]. 

The aim of the present study was (a) to compare BMI of MS patients presenting to a neurologic clinic, diagnosed as having clinically isolated syndrome (CIS) or relapsing-remitting MS, to BMI of subjects free from any major neurological disease and (b) to search for possible associations of the CSF levels of the main metabolites of noradrenaline, serotonin, and dopamine in patients who were drug-free at assessment with BMI.

## 2. Subjects and Methods

Four hundred and sixty-nine patients (154 males and 315 females) satisfying the revised McDonald criteria for MS or possible MS [21] and 358 controls (146 males and 212 females), within the age range of 18 to 60 years, were included in the study. Two hundred and sixty patients (55.4%), 83 males and 177 females, were drug-free at assessment. Patients on medication were included in comparing BMI in order to have a more representative sample, and possible influences of drug treatment on BMI were statistically evaluated. Only a small number of subjects, who at the time of evaluation were on treatment with atypical neuroleptics that are expected to strongly influence body weight [22], were not included in the study. 

Two hundred and ten patients (75 males and 135 females) had clinically isolated syndrome (CIS) suggestive of MS, and 259 patients (79 males and 180 females) had relapsing-remitting MS. Duration of illness ranged from 0.1 to 480 months, and score in the Expanded Disability Status Scale (EDSS) ranged from zero to 6.0. 

A control group in the same age range to the patients (18 to 60 years) was built from (a) 194 subjects (90 males and 104 females), admitted to the hospital for diagnostic investigations and found not to suffer from any major neurological disease, and (b) 164 healthy subjects (56 males and 108 females). The data of these two subgroups were merged after it was found that there were no differences in BMI (26.1 ± 4.0 and 26.7 ± 4.2 for males and 25.0 ± 4.5 and 25.8 ± 4.8 for females), to build a control group of 146 males and 212 females. Written informed consent was obtained from all subjects, and the study was approved by the Ethics Committee of the hospital.

Lumbar puncture was performed for diagnostic reasons in the lateral recumbent position between L3 and L4 or L4 and L5. The CSF fraction from the third to fifth milliliter was used for estimation of the metabolites. It was centrifuged at 1500 g for 15 min to remove cells and kept at −30°C until analysis, which was performed within 3 months from sampling. 

The levels of the neurotransmitter metabolites were estimated by HPLC with electrochemical detection, all three in the same run. A 4.6 mm column was used (Spherisorb ODS2, Waters, Milford, MA, USA), and the mobile phase consisted of acetate buffer, pH 5.2, with 10% methanol. For each sample an aliquot of CSF was directly injected into the HPLC system and subsequently a second aliquot with added standards corresponding to 5 ng/mL for MHPG, 10 ng/mL for 5-HIAA, and 20 ng/mL for HVA in the CSF sample. The concentrations of the metabolites were calculated from the differences in peak heights between the sample with and the sample without standards. For this part of the study, we only included patients who were drug-free for at least two weeks at the time of lumbar puncture, namely, 89 from the 145 males and 158 from the 285 females. 

Body mass index (weight in kilograms divided by the square of height in meters) was calculated using self-reported height and weight. Smoking history and numbers of cigarettes per day were registered. 

A multiple regression analysis was performed in searching for associations of clinical variables with BMI. Patients were assigned in subgroups regarding smoking (zero, 5–20, and >20 cigarettes per day), disease phenotype (CIS, RRMS), duration of illness (duration up to 3 months, 3 months to 3 years, and more than 3 years), and EDSS score (score zero, 1–3, and 3.5–6.5). Regarding treatment at assessment, patients were assigned in subgroups taking thyroid hormones (*n* = 29), steroids (*n* = 53), immunomodulatory agents (*n* = 20), antidepressives (*n* = 22), other drugs (*n* = 34), and drug-free patients (*n* = 301). Previous treatments with steroids (*n* = 199) and with immunomodulatory drugs (*n* = 58) were also used as independent variables. 

Since there were significant differences in weight, height, and BMI between genders in MS patients and in control subjects, the data were further analyzed separately for males and females. 

Quartile limits for weight, height, and BMI were calculated from male and female control subjects, and patients were thus assigned to defined quartiles. The differences of frequencies in lower, two middle, and upper quartiles between controls and patients were evaluated using the chi-square test. The CSF levels of MHPG, 5-HIAA, and HVA of drug-free patients assigned to BMI quartiles were compared separately for males and females, using analysis of variance with age as covariate.

## 3. Results

A multiple regression analysis was performed for the whole patient population (459 patients), with dependent variable BMI and independent variables sex, age, smoking, disease phenotype, duration of illness, drug treatment, EDSS score, previous treatment with steroids, and immunomodulatory drugs. A regression coefficient *R* = .3358 was calculated, with *F* = 6.34, df = 9, 449, and *P* < .0001. Significant associations with BMI were found only for sex (beta = −0.2293, *P* < .0001) and age (beta = 0.2103, *P* < .0001). For the total patients' samples, there were no associations of BMI with smoking, disease phenotype, duration of illness, drug treatment, previous treatment, or EDSS score. Moreover, Spearman correlation coefficient tests did not reveal any significant associations of BMI or weight with duration of illness, cigarettes smoked per day, or EDSS score. 

The data for age, weight, height, and BMI for male and female control subjects and MS patients and their evaluation using ANOVA with age as covariate are presented in Table 1. Body mass index was higher in males than in females, both in controls (*P* = .04) and patients with MS (*P* = .0001). 

Compared to male controls, male patients did not show any significant difference for weight, height, or BMI (Table 1). It is worth to mention, though, the near significance differences in height between CIS and controls (*P* = .054). The group of 310 female patients showed, compared to female controls, near significance (*P* = .09) lower body weight, significantly greater height (*P* = .006), and consequently lower BMI (*P* = .005). When disease phenotypes were compared to those in control subjects, these differences were found only for patients with RRMS. 

The frequencies of control subjects and MS patients in BMI quartiles are shown in Table 2. Higher frequencies of patients with lower BMI values compared to controls were found for female patients with CIS (*P* = .032) or with RRMS (*P* = .0001). The difference in frequencies was also significant when female patients with CIS were compared to RRMS females (*P* = .007).

CSF levels of noradrenaline, serotonin, and dopamine metabolites in control-defined BMI quartiles for males and females are shown in Table 3. Analyses of variance with age as covariate gave significant differences for 5-HIAA and for HVA for females and near significance for males. Planned comparisons between quartiles revealed significantly lower 5-HIAA and HVA levels of patients in the lower quartile compared to patients in the upper quartile, both for males (*P* = .02 and *P* = .04, resp.) and females (*P* = .008 and *P* = .001). No differences were found regarding the noradrenaline metabolite MHPG.

## 4. Discussion

For the female patient population, significantly lower BMI was found compared to that of sex and age matched controls. BMI was not related to duration of disease or EDSS score, but the fact that the difference from controls was not significant for subjects with CIS indicates that lower BMI is not present at disease onset but develops later, during disease progression. This highly significant difference (*P* < .005) in BMI is the result of higher than expected—compared to controls—numbers of patients with lower weight and also with greater height. For male patients, the difference in BMI was not significant. There was, however, a shift towards greater body height in patients compared to controls. 

Our results regarding BMI are in line with those of Nortvedt et al. [8], who reported for a mixed population of 22 males and 65 females (75% females) a BMI of 23.5 ± 3.6, with 11% of subjects having BMI below 20. In the present study of 149 males and 310 females (69% females), we calculated for the total sample a BMI of 24.2 ± 4.5, while 13.6% patients had BMI below 20.

Lower BMI in MS patients compared to age matched controls was previously reported also by Ghadirian et al. [3]. In their study, the difference in BMI was significant for the whole sample of 61 male and 136 female MS patients compared to 64 male and 138 female controls, but it did not reach statistical significance for males. Similar results were found in the present study. While BMI was significantly lower in female patients than controls, the difference for the male population did not reach significance.

An intriguing finding of this study is that body height of patients with MS tends to be greater than that of controls (Table 1). Similar findings were reported by Ghadirian et al. [3]. This finding is of specific interest within the context of the recent rise in the incidence of MS, observed in several countries. Increases in height over the last decades have been observed in most industrialized countries presumably as a result of better nutrition. It is also notable that the incidence of MS seems to be increasing among women but not men, with consequent increases in the female to male sex ratio [23, 24]. Another point that needs further investigation in MS is the connection of height growth with autoimmunity, as has been suggested for diabetes in children [25]. Since height is a highly heritable trait [26], it would be of interest to compare the height of patients to the target height calculated from heights of both parents [27], but such data were not collected in the present study.

A limitation of the current study is the self-reported weight and height that may not be accurate. This issue has been thoroughly studied by Stommel and Schoenborn [28]. They found that deviations of the self-reported from measured BMI values depend on many sociodemographic characteristics, among them sex and age. The most accurate values are obtained from middle-aged subjects, and deviations are greater at the high and low ends of the BMI scale. Importantly, self-reported and measured height and weight were highly correlated. The above mentioned factors are expected to apply for both patients and controls of the present case-control study and, taking also into account the high correlation between self-reported and measured values, are not expected to influence our results. We consider that the reliability of our results is increased by using as controls subjects presenting to the same neurology clinic during the same time period (several years). In addition, the results of the present study can be better compared to other studies that used also self-reported data for BMI [3, 9–13]. 

Male and female patients of the lower quartile for BMI were found to have lower CSF 5-HIAA and HVA levels compared to patients of the upper quartile (Table 3). The role of dopamine in energizing feeding and food-seeking behavior through its participation in brain reward circuits has been recognized [18, 29]. In addition, Kranz et al. [30] reviewing electrophysiological, pharmacological, genetic, and imaging studies postulated a role for serotonin in emotional, motivational, and cognitive aspects of reward representation, possibly as important as that for dopamine. The lower levels of HVA and 5-HIAA found in the present study in patients of the lower BMI quartile compared to patients in the upper quartile indicate that dopamine and serotonin may be implicated in food-seeking behavior in MS patients. Further research on motivation to eat and food reward capacity may show impairments in subjects predisposed to MS. Food reward elevates dopamine levels [16], and the low CSF HVA of subjects in the lower BMI quartile may represent an impaired dopamine reward system in MS patients. It has to be mentioned that impairments in neurotransmitter activities that influence eating behavior may exist with no effect on appearance or intensity of symptoms that are considered in scales that evaluate disability, for which other neural systems are responsible.

It is suggested that humoral factors from the periphery influence the dopaminergic system and affect food intake, either stimulating, like ghrelin, or inhibiting dopamine signaling, like leptin or insulin [15]. Serum leptin levels correlate positively to percentage of body fat and to BMI and are higher in women than in men, both in normal-weight and obese subjects [31]. Leptin signals nutritional status to other physiological systems and modulates their function [32]. When administered to animals, it reduces the activation of dopaminergic neurons and decreases food intake [33]. Increased leptin levels were found in CSF and serum of treatment-naïve RR MS patients compared to controls matched for age, gender, and BMI [34]. It can be hypothesized that in MS patients, especially in females, increased leptin production reduces dopamine signaling, thus attenuating the food reward circuits in the brain.

Payne [35] has reviewed the impact of nutrition on the aetiology and the rate of disease progression in MS. He concludes that there is no evidence that nutrition is involved in the aetiology of MS, while it is unclear whether diet may influence disease progression, although malnutrition may contribute to disability, since it is related to muscle weakness and fatigue. In a recent case-control study, Hedstrom et al. [13] reported no differences in BMI between MS patients and controls but found that subjects who reported body weight at age 20 that gave a BMI over 25 had higher risk for developing MS. This does not necessarily mean that losing weight may be beneficial, if the weight at disease onset is not known. Deficits in serotoninergic and dopaminergic turnovers arise during disease course [20], and this may influence food reward capacity, facilitating reductions in body weight. The low levels of serotonin and dopamine metabolites of subjects in the lower BMI quartile found in the present study support this assumption. Our results indicate the need for further studies with information on BMI at adolescence, at first episode, and later at definite MS before it can be concluded that overweight at adolescence is a predisposing factor for MS, and losing weight may be beneficial.

## Figures and Tables

**Table 1:** Age, weight (kg), height (cm), and body mass index (means ± SD) in male and female control subjects and patients with MS. Duration of illness (months) and EDSS score are given for patient groups.

Group	*N*	Age	Weight	Height	BMI	Duration	EDSS
Males							
Control	146	35.9 ± 7.5	81.5 ± 14.6	176 ± 8	26.3 ± 4.1		
CIS + RRMS	154	33.8 ± 9.3	81.6 ± 12.5	178 ± 7	25.7 ± 3.3	43.5 ± 77.0	1.9 ± 1.1
CIS	75	32.9 ± 8.8	81.4 ± 11.9	179 ± 7*	25.5 ± 3.2	5.0 ± 10.1	1.7 ± 1.0
RRMS	79	34.7 ± 9.9	81.8 ± 13.2	177 ± 7	25.9 ± 3.4	80.0 ± 93.6	2.2 ± 1.2
Females							
Control	212	36.9 ± 9.4	68.0 ± 13.4	163 ± 6	25.5 ± 4.6^#^		
CIS + RRMS	315	35.2 ± 9.6	64.3 ± 13.7*	166 ± 6**	23.4 ± 4.8^#∗∗^	42.5 ± 58.5	1.7 ± 1.2
CIS	135	36.0 ± 10.1	66.1 ± 15.1	165 ± 5*	24.2 ± 5.1*	10.2 ± 31.6	1.3 ± 1.1
RRMS	180	34.6 ± 9.2	63.0 ± 12.4*	166 ± 6**	22.9 ± 4.4**	66.7 ± 62.7	1.9 ± 1.2

CIS: clinically isolated syndrome suggesting of MS; RR: Relapsing-Remitting MS. ^#^Significantly lower compared to males.  _ _*P < 0.05  and  _ _**P < 0.01, compared to same sex control group (ANOVA with age as covariate).

**Table 2:** Number of subjects in BMI quartiles defined from male controls (BMI limits 23.51 and 27.90) and female controls (BMI limits 21.81 and 28.58). Percent of subjects in parentheses.

	Males			Females		
	Lower	2nd + 3rd	Upper	Lower	2nd + 3rd	Upper
Controls	37 (25.3)	73 (50.0)	36 (24.7)	53 (25.0)	105 (50.9)	51 (24.1)
CIS	22 (29.3)	38 (50.7)	15 (20.0)	45 (33.3)	72 (53.3)	18 (13.4)
RRMS	20 (25.3)	42 (53.2)	17 (21.5)	92 (51.1)	71 (39.4)	17 (9.4)

	Chi-square		*P*	Chi-square		*P*

CNTR versus CIS	0.77		.68	6.89		.032
CNTR versus RRMS	0.35		.85	32.74		.0001
CIS versus RRMS	0.32		.85	9.93		.007

**Table 3:** Levels (ng/mL) of noradrenaline (MHPG), serotonin (5-HIAA), and dopamine (HVA) metabolites in CSF of drug-free multiple sclerosis patients, grouped in quartiles according to their body mass index. Quartile limits were defined from BMI of control subjects of the present study, separate for males and for females. Statistical evaluation by analysis of variance with age as covariate.

BMI quartile	*N*	BMI	Age	MHPG	5-HIAA	HVA
Male patients						
Lower	23	22.3 ± 1.0	32.2 ± 8.4	6.64 ± 1.85	12.7 ± 5.9	22.6 ± 8.3
Middle (2nd + 3rd)	46	25.7 ± 1.2	34.8 ± 8.5	6.16 ± 1.97	14.6 ± 7.1	24.7 ± 11.4
Upper	14	30.4 ± 2.2	32.7 ± 5.2	6.83 ± 2.68	15.4 ± 10.5	26.0 ± 12.9
*F* (2, 79)				0.67	0.76	0.54
*P*				.52	.47	.58
Female patients						
Lower	77	19.9 ± 1.3	32.2 ± 7.9	6.37 ± 1.84	15.6 ± 5.6	25.8 ± 9.1
Middle (2nd + 3rd)	79	24.2 ± 1.6	36.6 ± 9.8	6.69 ± 1.93	18.3 ± 7.9	31.3 ± 11.6
Upper	21	33.8 ± 4.4	38.9 ± 11.0	7.08 ± 2.25	21.5 ± 5.9	36.6 ± 16.1
*F* (2, 173)				0.85	5.05	9.50
*P*				.43	.007	.0001
Lower versus upper, *P*				.21	.003	.0001
